# NLRX1/FUNDC1/NIPSNAP1‐2 axis regulates mitophagy and alleviates intestinal ischaemia/reperfusion injury

**DOI:** 10.1111/cpr.12986

**Published:** 2021-01-11

**Authors:** Shaoqin Li, Yi Zhou, Xiaocheng Gu, Xiaoping Zhang, Zhongzhi Jia

**Affiliations:** ^1^ Department of Interventional and Vascular Surgery Changzhou No. 2 People's Hospital Changzhou China; ^2^ Department of Nuclear Medicine Shanghai Tenth People's Hospital Tongji University Shanghai China; ^3^ Shanghai Center of Thyroid Diseases Tongji University School of Medicine Shanghai China

**Keywords:** FUNDC1, hypoxia reoxygenation injury, ischaemia reperfusion injury, mitophagy, NIPSNAP1‐2, NLRX1

## Abstract

**Objectives:**

Mitophagy is considered to be a key mechanism in the pathogenesis of intestinal ischaemic reperfusion (IR) injury. NOD‐like receptor X1 (NLRX1) is located in the mitochondria and is highly expressed in the intestine, and is known to modulate ROS production, mitochondrial damage, autophagy and apoptosis. However, the function of NLRX1 in intestinal IR injury is unclear.

**Materials and methods:**

NLRX1 in rats with IR injury or in IEC‐6 cells with hypoxia reoxygenation (HR) injury were measured by Western blotting, real‐time PCR and immunohistochemistry. The function of NLRX1‐FUNDC1‐NIPSNAP1/NIPSNAP2 axis in mitochondrial homeostasis and cell apoptosis were assessed in vitro.

**Results:**

NLRX1 is significantly downregulated following intestinal IR injury. In vivo studies showed that rats overexpressing NLRX1 exhibited resistance against intestinal IR injury and mitochondrial dysfunction. These beneficial effects of NLRX1 overexpression were dependent on mitophagy activation. Functional studies showed that HR injury reduced NLRX1 expression, which promoted phosphorylation of FUN14 domain‐containing 1 (FUNDC1). Based on immunoprecipitation studies, it was evident that phosphorylated FUNDC1 could not interact with the mitophagy signalling proteins NIPSNAP1 and NIPSNAP2 on the outer membrane of damaged mitochondria, which failed to launch the mitophagy process, resulting in the accumulation of damaged mitochondria and epithelial apoptosis.

**Conclusions:**

NLRX1 regulates mitophagy via FUNDC1‐NIPSNAP1/NIPSNAP2 signalling pathway. Thus, this study provides a potential target for the development of a therapeutic strategy for intestinal IR injury.

## Graphical abstract

The overexpression of NLRX1 in IEC‐6 cell under hypoxia reoxygenation injury, which inhibited phosphorylation of FUN14 domain‐containing 1 (FUNDC1). Based on immunoprecipitation studies, it was evident that dephosphorylated FUNDC1 could interact with the mitophagy signalling proteins NIPSNAP1 and NIPSNAP2 on the outer membrane of damaged mitochondria, which launch the mitophagy process, reduced the accumulation of damaged mitochondria and epithelial apoptosis.

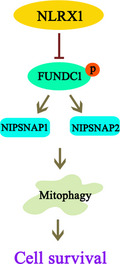

## 1 INTRODUCTION

Ischaemic reperfusion (IR) injury is characterized as the damage caused to the tissues due to the return of blood supply (reperfusion) after a brief duration of lack of oxygen (ischaemia). Affecting a wide range of population and organs, they are usually observed as a consequence to conditions such as trauma, shock, strangulation, mesenteric artery thrombosis and intestinal obstruction.^1^, ^2^, ^3^, ^4^ Ischaemia by itself damages the cells, which is further exacerbated by the restoration of blood flow resulting in increased cell death and release of inflammatory response elements that ultimately leads to multiple organ failure.^5^ Of the organs affected by IR injury, the intestine has been described as the most vulnerable to this damage and severe intestinal injury could lead to the need for immediate surgical removal of intestinal regions.^6^, ^7^ Additionally, without appropriate diagnosis or treatment, risk of increased mortality and morbidity becomes exponentially high.^8^

Mitochondrial autophagy (mitophagy) is an important process which eliminates damaged mitochondria and maintains mitochondrial homeostasis.^9^ Previously, many studies have associated defects in mitophagy to IR injury, where in increased accumulation of damaged mitochondria has been suggested to lead to mitochondrial dysfunction and increased apoptosis.^9^, ^10^ Some studies also indicate that mitochondrial dysfunction could be one of the leading cause of the increase in ROS production and thus in turn contributing to the increase in apoptosis.^11^, ^12^ Even though many evidences have indicated the consequences of mitochondrial dysfunction in IR injury, there is still a strong need to elucidate the cause of mitochondrial dysfunction specifically in intestinal IR injury.

NOD‐like receptor X1 (NLRX1) is a member of the NLR family of proteins and is well known as a regulator of the immune system. It has been identified to be predominantly localized in the mitochondria and is highly expressed in the intestine.^13^, ^14^ Interestingly, with varied functions such as, negative regulation of the NF‐κb, IFN‐1, JNK and MAPK signalling pathways; modulation of ROS production; regulation of mitochondrial damage, autophagy and apoptosis; NLRX1 has been characterized as an enigmatic player in the field of molecular biology.^13^, ^14^ In this study, we identified that NLRX1 levels were significantly decreased after IR. Another key player in mitophagy is FUN14 domain‐containing 1 (FUNDC1). FUNDC1 is a mitochondrial membrane protein that interacts with autophagy members to increase mitophagosomes and thus maintain appropriate mitochondrial homeostasis.^15^, ^16^ FUNDC1‐related mitophagy dysfunction has been associated with many cardiovascular and metabolic diseases, as FUNDC1 has been identified to protect the cells from hypoxia‐associated damage.^17^, ^18^, ^19^ However, FUNDC1’s role in intestinal IR has not been well understood. Interestingly, in our study, we found that reduced NLRX1 was associated with increased phosphorylation of FUNDC1 under intestinal IR injury.

Another key member of the mitophagy process is the nitrophenylphosphatase domain and non‐neuronal SNAP25‐like protein homolog 1 and 2 (NIPSNAP 1 and 2) which upon depolarization of the mitochondrial membrane accumulate on the surface and recruit many autophagy associated proteins thus acting as an ‘eat me’ signal.^20^, ^21^ Hence, appropriate NIPSNAP1 and 2 signalling are required for the maintenance of mitochondrial homeostasis. In the current study, we report that NLRX1 is significantly downregulated following intestinal IR injury, and its level is negatively correlated with epithelial cell apoptosis and mitochondrial damage. In vivo studies indicated that overexpression of NLRX1 alleviates IR injury through activation of mitophagy. Functional studies using in vitro models showed that HR injury reduced NLRX1 expression, which promoted phosphorylation of FUN14 domain‐containing 1 (FUNDC1) and thus failed to interact and activate NIPSNAP1 and NIPSNAP2. Based on these findings, it is evident that NLRX1 regulates mitophagy via FUNDC1‐NIPSNAP1/NIPSNAP2 signalling pathway.

## 2 MATERIALS AND METHODS

### 2.1 Animal model of intestinal IR injury

The animal study was reviewed and approved by the Animal Care and Use Committee of Changzhou No. 2 People's Hospital. For the development of intestinal IR injury model, eight‐week‐old Male Sprague Dawley rats (weighing 200‐220 g) were purchased from Shanghai slac Laboratory Animal Ltd., China. Rats were randomly assigned into four groups (n = 8 in each group). The sham group rats were intragastrically administered saline and underwent isolation of superior mesenteric artery without clamping. Intestinal ischaemia in rats was induced by following the protocol of a previously published study.^22^ Briefly, after a pre‐requisite 24 hours fasting, animals were intraperitoneally injected with 50 mg/kg of sodium pentobarbital (Sigma‐Aldrich). Ischaemic injury of the intestine was further achieved by clamping of superior mesenteric artery. Further, intestine was reperfused after 45 minutes for varying time periods as indicated in the results. In some cases, rats were overexpressed using adenovirus encoding NLRX1 (by Gene Pharma Corporation) along with respective controls.

### 2.2 Cell culture, hypoxia/reoxygenation (HR) and treatment

IEC‐6 cells were cultured in DMEM (Invitrogen, Carlsbad, CA, USA) with 10% FBS, 1% P/S (Sigma‐Aldrich) and maintained in 5% CO_2_ at 37°C. To induce hypoxia, we placed cells in a hypoxic chamber with 5% CO_2_, 94% N_2_ and 1% O_2_ at 37°C for 1 hour, and this was followed by reoxygenation for 0, 0.75, 3 or 6 hours. A recombinant adenovirus expressing NLRX1, FUNDC1 siRNA and vehicle were designed and chemically synthesized by Gene Pharma Corporation (Shanghai, China) and transfected into the cells using lipofectamine. To inhibit mitophagy, the cells were treated with 3‐MA (10 mmol/L) for 2 hours.

To achieve NLRX1 overexpression, the pDC316‐mCMV‐NLRX1 plasmid was purchased from Gene Pharma Corporation (Shanghai, China) and subsequently transfected with a framework plasmid (1:1) into 293 T cells using Lipofectamine 2000. After transfection for 48 hours, the viral supernatant was collected and assessed by PCR. Following amplification, the supernatant was collected again and filtered through a 0.45 μm filter to obtain adenovirus‐NLRX1 (Ad‐NLRX1). Thereafter, IEC‐6 cells were infected with Ad‐NLRX1 for 6 hours at 37°C/5% CO_2_. The media were then replaced with fresh culture medium. After 24 hours culture, cells were washed with PBS and collected for experimentation. Western blot was used to evaluate the overexpression efficiency.

### 2.3 Quantitative real‐time polymerase chain reaction and biochemical analysis

Using RNeasy Mini Kit (Qiagen, Hamburg, Germany), total RNA was isolated from the samples. Further, 2 μg of RNA was reverse transcribed using PrimeScript™ RT reagent kit. Further, quantitative real‐time PCR was performed according to a previous study.^23^ Primer sequences and PCR settings were as follows: IL‐1β: forward, 5′‐ACGCTTACCATGTGAGCTG‐3′, reverse, 5′‐GCCACAGGGATTTTGTCGTT‐3′; IL‐6: forward, 5′‐ACGCTTCTGGGCCTGTTGTT‐3′, reverse, 5′‐CCTGCTGCTGGTGATTCTCT‐3′; TNF‐α: forward, 5′‐TCGGTCCCAACAAGGAGGAG‐3′, reverse, 5′‐GGGTTGTCACTCGAGTTTTG‐3′; MCP1: forward, 5′‐CTCAGCCAGATGCAATCAAT‐3′, reverse, 5′‐GCTTCTTTGGGACACTTGCT‐3′; and Gapdh: forward, 5′‐GCCAAGGCTGTGGGCAAGGT‐3′, reverse, 5′‐TCTCCAGGCGGCACGCAGA‐3′.

To assess the levels of MDA, SOD, GSH and MPO, rats were sacrificed and the intestinal tissue was excised, homogenized and dissolved in extraction buffer.^24^ Further, the samples were centrifuged at 6000 RPM for 15 minutes and the supernatant fraction were analysed for MDA, SOD, GSH and MPO levels using commercial kits (Biotechnology, Nanjing, China) according to the manufacturer’s instructions.

The ALT and AST in the serum were also assessed using commercial kits (Biotechnology, Nanjing, China).

### 2.4 Histopathological analysis

Post‐sacrifice of the animals, ileum samples were isolated and fixed with 4% paraformaldehyde for at 24 hours. Further, tissue blocks were cut into 5‐µm slices, stained with haematoxylin and eosin (H&E) and analysed under a light microscope (Leica DM4000B, Germany).

### 2.5 Western blot analysis and co‐immunoprecipitation

Total protein was either isolated from the animal intestinal tissues or IEC‐6 cells using total protein extraction kit according to the manufacturer’s instruction and quantified using Bradford assay (ab102535; Abcam). Protein samples at a concentration of 25 μg were separated using 10%‐25% sodium dodecyl sulphate (SDS) gel and transferred onto a PVDF membrane (Millipore). Blocking of the membranes was performed using 5% skim milk. The membranes were further incubated with respective primary antibody overnight. The following primary antibodies were used for Western blot analyses: NLRX1 (1:3000), LC3I/II (1:1000), LC3II (1:1000), p62 (1:1000), Tim23 (1:1000), cleaved caspase 3 (1:1000), caspase‐9 (1:1000) and Tomm20 (1:1000) were purchased from Abcam; NIPSNAP1 (1:1000), NIPSNAP2 (1:1000), cytochrome‐c (1:1000), Parkin (1:1000), ZO‐1 (1:500), occludin (1:500) and GAPDH (1:1000) were purchased from Cell Signaling Technology. Anti‐p‐FUNDC1 (Tyr18) (1:500) and anti‐FUNDC1 (1:1000) polyclonal antibodies were produced by immunizing rabbits with synthesized and purified phosphorylated and non‐phosphorylated peptides from FUNDC1 (Abgent, Suzhou, China) according to a previous study.^23^ Membranes were washed thrice using PBS‐T. Further, the membranes were incubated in respective secondary antibodies for 1 h and visualized using an ECL detection system. Quantification of the protein bands were carried out using ImageJ software (US National Institutes of Health, Bethesda, MD, USA) and normalized using GAPDH levels.

Co‐immunoprecipitation experiments were performed as previously described.^25^ Briefly, proteins from cells were cross‐linked in 1% paraformaldehyde followed by washing in PBS containing 100 mmol/L glycine. The cells were then lysed by sonication in PBS with 1% Triton X‐100 and incubated with the respective antibodies and protein A/G agarose. The immunoprecipitated samples were loaded onto SDS‐PAGE gel and probed with FUNDC1 antibody.

### 2.6 Cell viability and apoptotic cell death assay

To assess the IEC‐6 cells viability, CCK‐8 assay (ab228554; Abcam) was performed based on the manufacturer’s instruction. TUNEL assay (Roche Applied Bio Sciences) (in vitro and vivo) were conducted to detect cellular apoptosis according to the manufacturer’s instructions.

### 2.7 Measurement of intracellular ROS levels

ROS levels were measured using chloromethyldihydrodichlorofluorescein diacetate (CM‐H2DCF/DA) assay by ROS Detection Cell‐Based Assay Kit (Item# 601520, CAYMAN CHEMICAL) according to the manufacturer’s protocol.

### 2.8 Mitochondrial membrane potentials assay

IEC‐6 cells were seeded onto a 6‐well dish and cultured for 24 hours at 37°C with 5% CO_2_. The ΔΨm was visualized using the JC‐1 Kit following the manufacturer’s instructions (Beyotime, China). Briefly, the cells were washed initially with dilution buffer and stained with 20 μmol/L JC‐1 dye and incubated in 37°C for 10 minutes. Further, the cells were washed using 1× dilution buffer, and analysed under a light microscope.

### 2.9 Transmission Electron Microscopy (TEM)

To assess mitophagy using TEM, a previously published protocol was followed.^26^ Briefly, IEC‐6 cells were seeded onto coverslips and cultured in 24‐well dish for 24 hours at 37°C in the presence of 5% CO_2_. Further, the cells were fixed using 4% glutaraldehyde and then treated with 2% osmium tetroxide. The cells were embedded into epoxy resin after dehydration in graded ethanol. Ultra‐thin sections were cut using an ultra microtome and stained using lead citrate. The structural mitophagy was assessed and observed using TEM.

### 2.10 Immunofluorescence and immunohistochemistry

Immunofluorescence and immunohistochemistry were carried out using a previously published protocol.^27^ The tissues were embedded into OCT (Tissue‐Tek; Sakura Finetek U.S.A., Inc.) and into 7 μmol/L sections using a cryostat. Briefly, the cells and tissues sections were fixed using 4% paraformaldehyde for 15 minutes and permeabilized using 0.5% Triton x‐100 for 10 mins. Further, the cells were washed with PBS‐0.25% Tween‐20 (PBS‐T) thrice for 5 minutes each. Further, the cells or tissue sections were incubated with primary antibodies overnight at 4°C. The primary antibodies used in the present study were as follows: anti‐NLRX1 (1:3000), LC3II (1:1000), NIPSNAP1 (1:1000), NIPSNAP2 (1:1000), cytochrome‐c (1:1000), Tomm20, (mitochondrial antibody, 1:500, Abcam) and Lamp1 (lysosome antibody, 1:500, Abcam). The mitochondrial and lysosome antibodies were used to mark mitochondria and lysosomes, respectively.^28^ The tissue sections or cells were washed thoroughly using PBS‐T and incubated with respective secondary antibodies (Alexa Fluor 488; Invitro gen) for 1 hour. After washing with PBS‐T and staining with Hoechst (ab228550; Abcam), the sections and cells were visualized using a fluorescent microscope (Olympus FluoView™ FV1000, Tokyo, Japan).

### 2.11 Statistical analysis

Data are shown as the mean ± standard error of the mean (SEM). Each experiment was repeated three times. Student’s *t* test or one‐way ANOVA was used for statistical analysis using Prism 6 (GraphPad Software). *P* < .05 was considered statistically significant.

## 3 RESULTS

### 3.1 Down‐regulation of NLRX1 contributes to intestinal morphological damage after IR

**FIGURE 1:**
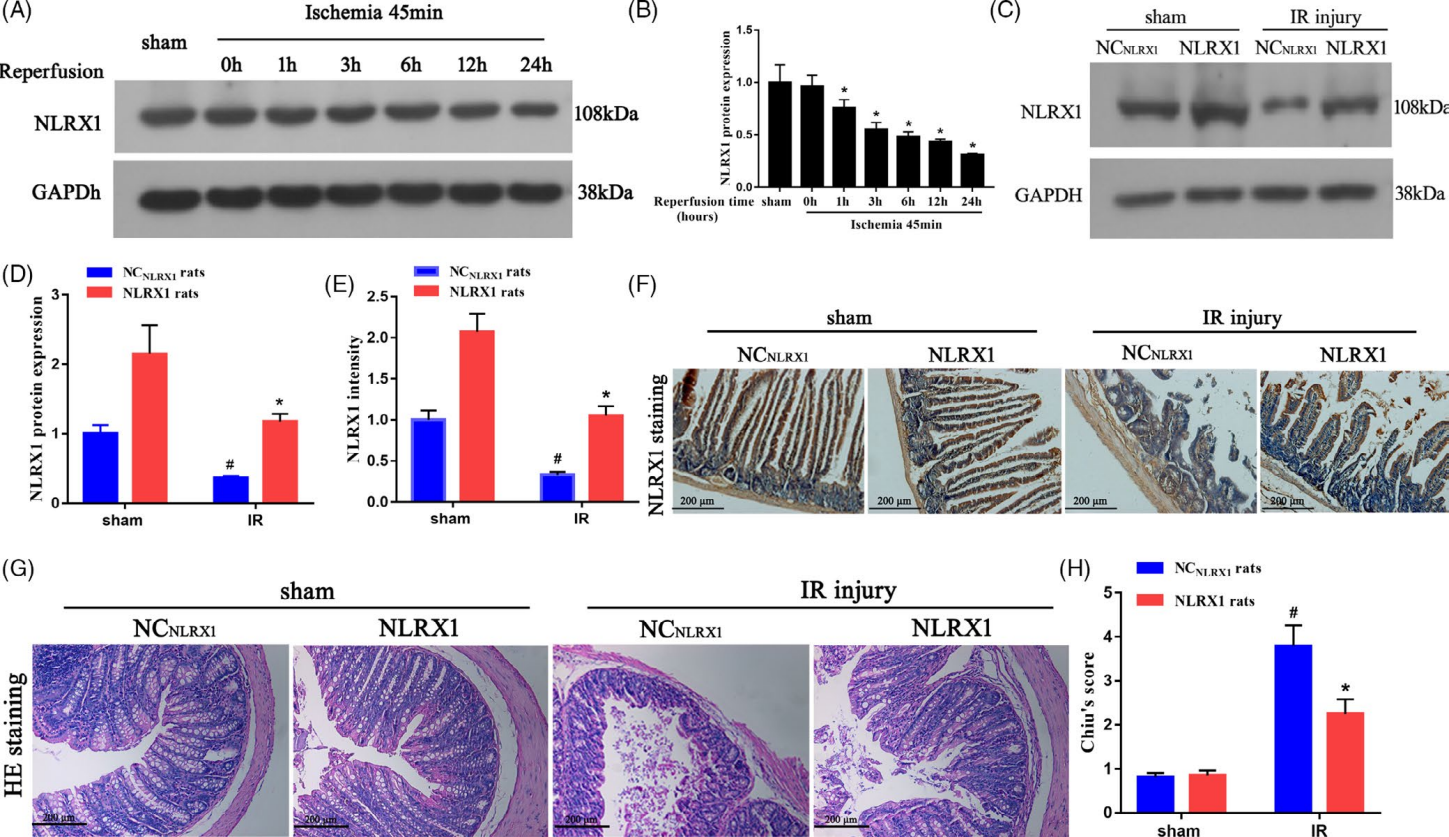
NLRX1 inhibits IR‐induced intestinal morphologic injury. A, B, Western blotting of the intestinal protein samples were carried out to assess the levels of NLRX1 before and after reperfusion. *P < .05 vs 0‐h reperfusion. C, D, Level of NLRX1 was verified using Western blotting in NLRX1 overexpression (NLRX1) rats. E, F, Immunohistochemistry assay of NLRX1 in control (NC_NLRX1_) and NLRX1 overexpression rats. (scale bar = 200 μm; magnification ×100). G, Representative morphologic changes in rat ileum (scale bar = 200 μm; magnification ×100) after 45 min of intestinal ischaemia followed by 24 h of reperfusion. H, Chiu’s scores were calculated as described in the Section 2. NC: negative control. Values are expressed as mean ± SEM (n = 8). *^#^P *< .05 vs sham group; *P < .05 vs NC_NLRX1_+IR group

Initially, to assess the role of NLRX1, eight‐week‐old male Sprague Dawley rats were subjected to intestinal ischaemia for 45 mins followed by reperfusion for 0, 1, 3, 6, 12 or 24 hours. We then performed Western blotting analysis of the intestinal samples and observed that as exposure time to IR increased, NLRX1 expression levels significantly decreased in rats (IR) (Figure 1A,B). Further, to confirm the role of NLRX1, we overexpressed NLRX1 in intestinal IR injury rat models. Western blots of NLRX1 demonstrated that NLRX1 was significantly decreased in ileum, and these changes were rescued in rats with NLRX1 overexpression (Figure 1C,D). This evidence was further visualized using immunohistochemistry, where we observed increased expression of NLRX1 along the ileum post‐overexpression of NLRX1 (Figure 1E,F). Intestinal damage associated with IR injury was evident in the control group (NC_NLRX1_), but overexpression of NLRX1 appeared to significantly decrease the intestinal morphological damage as indicated by the decrease in Chiu’s score (Figure 1G,H).

### 3.2 NLRX1 overexpression reduces intestinal IR‐induced oxidative stress and inflammatory response

**FIGURE 2:**
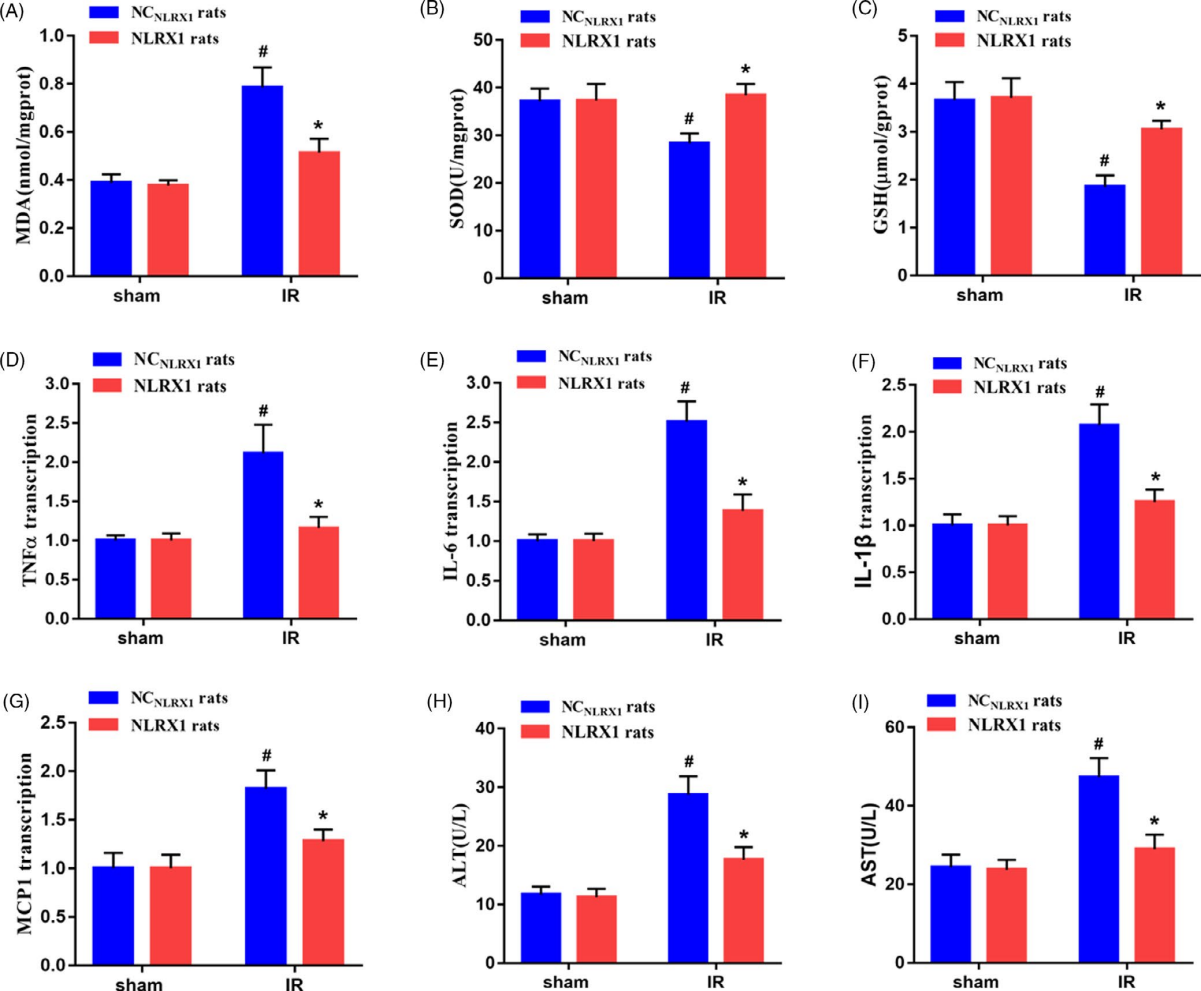
NLRX1‐induced inhibition of the inflammatory response and oxidative stress in intestinal IR injured rats. a‐g Effect of NLRX1 overexpression on (A) MDA, (B) SOD, (C) GSH, (D) TNFα, (E) IL‐6, (F) IL‐1β, (G) MCP1 in intestinal tissues (n = 8). H‐I, Effects of NLRX1 overexpression on serum (H) ALT and (I) AST levels (n = 8). Values are expressed as mean ± SEM (n = 8). *^#^P *< .05 vs sham group; *P < .05 vs NC_NLRX1_+IR group

To elucidate further the mechanistic role of NLRX1 on IR injury, we assessed the levels of inflammatory response and oxidative stress markers in the IR injured rat models. Initially, we observed that oxidative stress indicators malondialdehyde (MDA) was increased, whereas sodium dismutase (SOD) and glutathione (GSH) were decreased in the IR injury models. However, overexpression with NLRX1 seems to significantly decrease MDA and increase SOD and GSH in the NLRX1 models (Figure 2A‐C). Next, we checked the inflammatory markers and observed increased levels of tumour necrosis factor α (TNFα), interleukin‐6 (IL‐6), IL‐1β and monocyte chemoattractant protein‐1 (MCP‐1) in IR injured rats (Figure 2D‐G). Interestingly, we also observed increased levels of alanine transaminase (ALT) and aspartate transaminase (AST), which are the serum biochemical indicators of remote organ injury, suggesting that NLRX1 alleviates remote organs damage without significantly affecting these parameters in sham rats (Figure 2H,I). Further, overexpression with NLRX1 significantly decreased the levels of inflammatory and oxidative stress, thus indicating a potential rescue after IR injury.

### 3.3 Overexpression of NLRX1 promotes mitophagy activation and reduces intestinal IR‐induced injury

**FIGURE 3:**
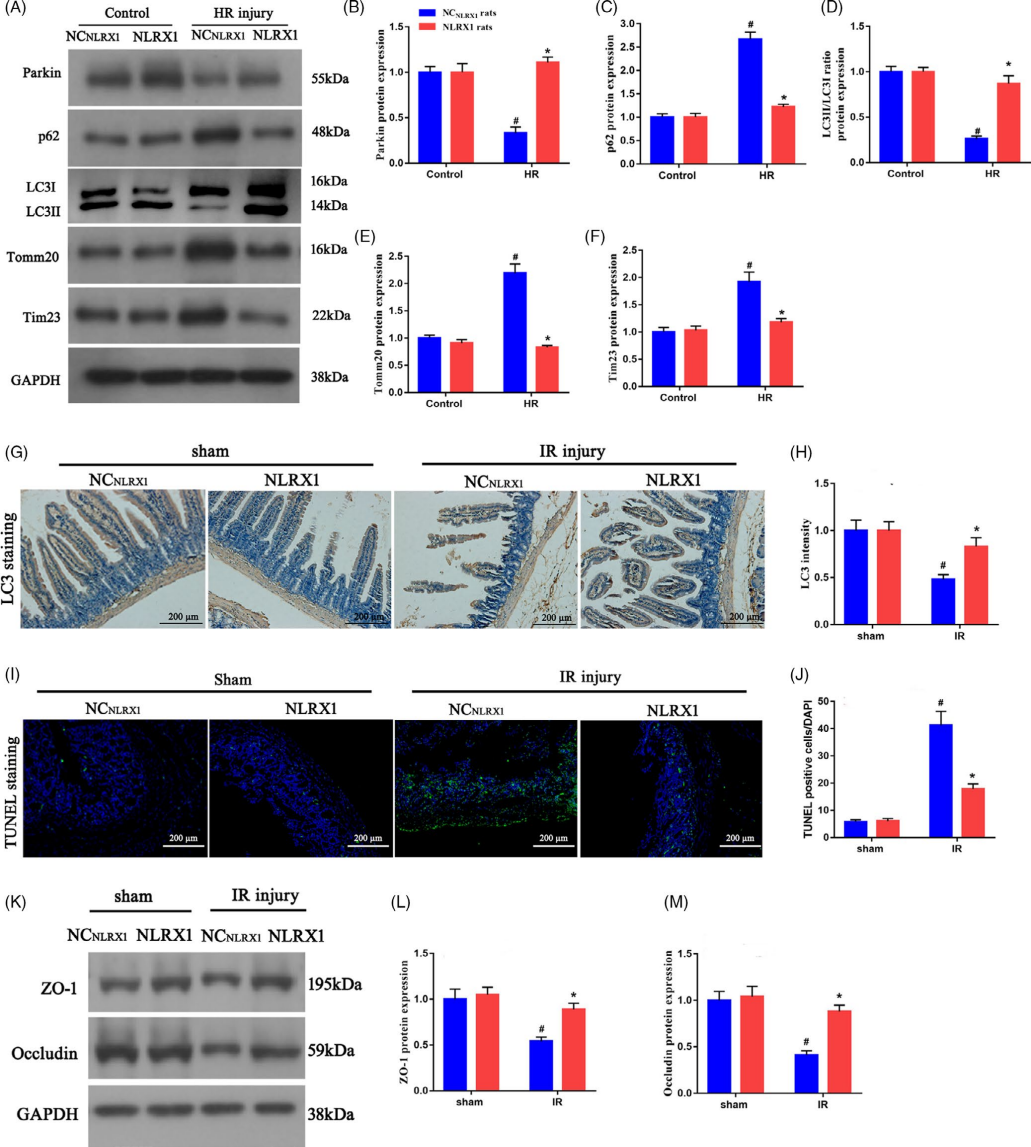
NLRX1 promotes mitophagy and alleviates intestinal IR‐induced injury. A‐F, Western blotting experiments to assess mitophagy flux (parkin, p62 and LC3II/LC3I ratio) and mitochondrial protein (Tomm20 and Tim23) levels in intestinal tissues. G,H, Immunohistochemistry assay of LCⅡ in NC_NLRX1_ and NLRX1 overexpression rats. (scale bar = 200 μm; magnification ×100). I,J, Representative TUNEL staining and quantitative analysis after intestinal IR injury. Relative apoptotic rates are represented as TUNEL‐positive cells/DAPI (scale bar = 200 μm; magnification ×100). K‐M, Protein expression levels of ZO‐1 and occludin. *^#^P *< .05 vs sham group; *P < .05 vs NC_NLRX1_+IR group

To establish the role of NLRX1 on mitophagy, we assessed the level of mitophagy‐associated proteins in the intestine of our in vivo models. Initially, it was evident that IR injury decreased mitophagy‐associated protein Parkin, however, overexpression of NLRX1 significantly increased Parkin levels. Further, we could observe an increase in mitochondria‐associated protein such as Tomm 20 and Tim 23 under IR injury. However, this could be again decreased in the presence of NLRX1. Next, we observed a significant increase in p62 levels and decrease in LC3‐II levels under IR injury. Overexpression of NLRX1 could revert this effect by decreasing p62 levels and increasing LC3‐II levels. These results do indicate that IR injury decreases mitophagy and overexpression of NLRX1 could rescue this defect (Figure 3A‐F). These results were further confirmed using LC3 staining of the IR injured rat models, where we observed increased levels of LC3 staining after NLRX1 overexpression in rat intestinal tissues (Figure 3G,H). Further, we also performed TUNEL staining and observed an increased level of apoptosis after IR injury in rat models, but this apoptosis could be significantly decreased with overexpression of NLRX1 (Figure 3I,J). We additionally performed Western blotting to assess the membrane proteins zonula occludens‐1 (ZO‐1) and occludin levels and observed that IR injury significantly decreased the membrane protein levels. We further overexpressed NLRX1 and observed a rescue and increase in the membrane protein levels (Figure 3K‐M). These results clearly indicate that IR injury decreases mitophagy and increases apoptosis, whereas NLRX1 could rescue this defect.

### 3.4 NLRX1 changes FUNDC1/NIPSNAP1‐2 protein expression in vitro

**FIGURE 4:**
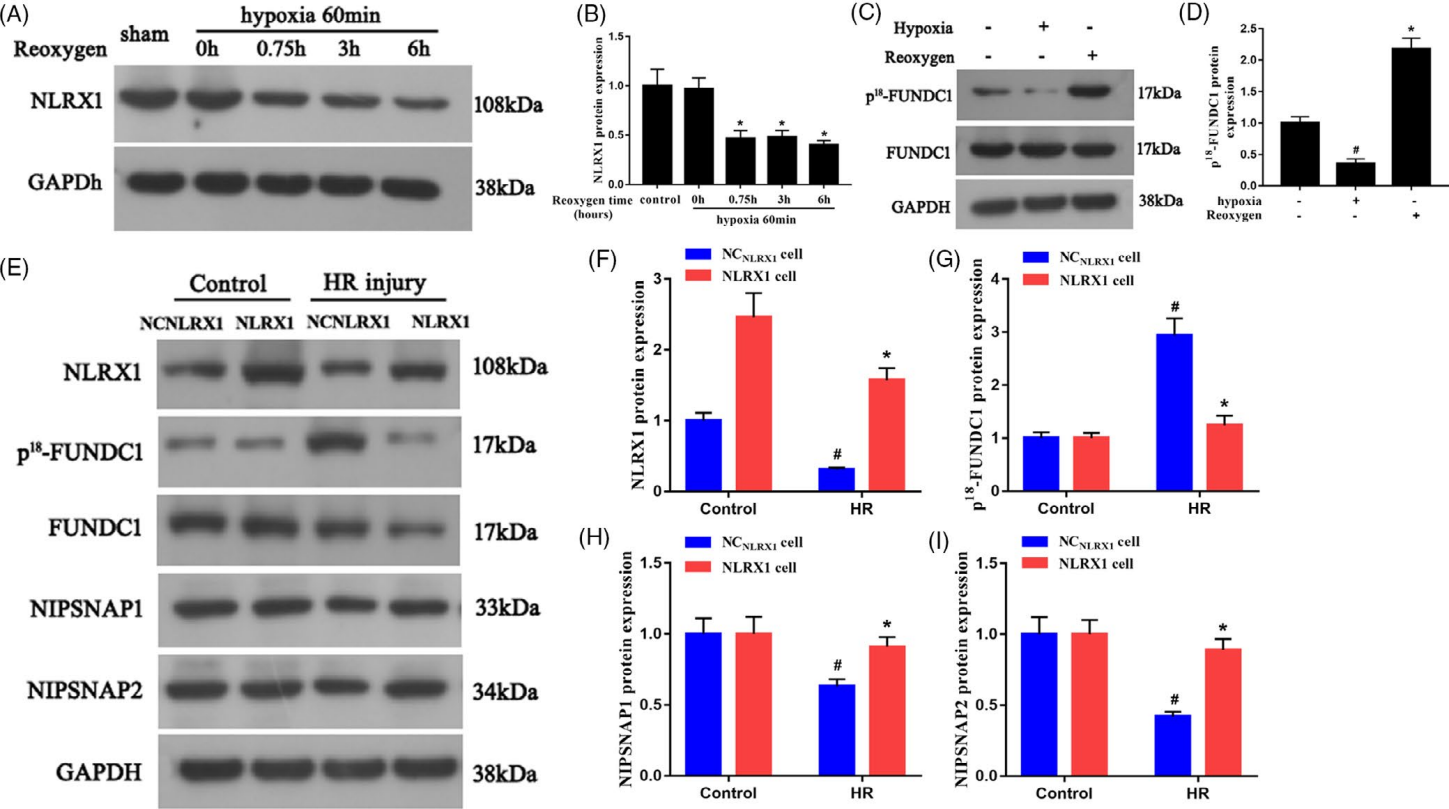
NLRX1 affects FUNDC1 and NIPSNAP1‐2 protein expression in IEC‐6 cells after HR. A,B, After HR, proteins were isolated and Western blotting was carried out to analyse the levels of NLRX1 before and after reoxygenation. *P < .05 vs 0‐h reoxygenation. C,D, The difference in change of p^18^‐FUNDC1 levels after hypoxia and reoxygenation. *^#^P* < .05 vs control; *P < .05 vs hypoxia. E‐I, The expression of NLRX1, p^18^‐FUNDC1, NIPSNAP1 and NIPSNAP2 in HR injury with either empty plasmid or NLRX1 overexpression plasmid. Overexpression of NLRX1 at the reoxygenation phase could reverse FUNDC1 activity. *^#^P*<.05 vs control group; *P < .05 vs NC_NLRX1_+HR group

Using IEC‐6 cells HR injury model, we further assessed the mechanistic aspect behind NLRX1’s influence on IR injury. Initially, we confirmed that HR injury significantly decreased NLRX1 levels in IEC‐6 cells (Figure 4A,B). As the expression levels of NLRX1 for 0.75, 3 and 6 hours did not change significantly, follow‐up experiments were performed using 0.75 hours of reperfusion. Further, we observed that hypoxia decreased the levels of Tyr18 phosphorylated FUN14 domain‐containing 1 (P^18^‐FUNDC1), and however, reoxygenation seemed to increase its levels significantly compared to the control (Figure 4C,D). Further, we overexpressed NLRX1 in the HR injury model and observed that decrease in NLRX1 levels corresponded to an increase in P^18^‐FUNDC1 levels, whereas overexpression with NLRX1 decreased the P^18^‐FUNDC1 levels (Figure 4E‐G). It was clear from the above‐mentioned results, that hypoxia activated FUNDC1 by dephosphorylation of Tyr 18 of FUNDC1, and however, reoxygenation seems to significantly increase its modification. Further, we checked the levels of mitophagy related proteins NIPSNAP1 and NIPSNAP2, and observed that HR injury decreased both the mitophagy proteins significantly (Figure 4E,H,I). However, overexpression with NLRX1 significantly increased NIPSNAP1 and NIPSNAP2 levels. These results indicated that NLRX1 regulates FUNDC1 and NIPSNAP1‐2 protein expression in IEC‐6 cells after HR.

### 3.5 NLRX1 inhibits mitochondrial apoptosis under HR

**FIGURE 5:**
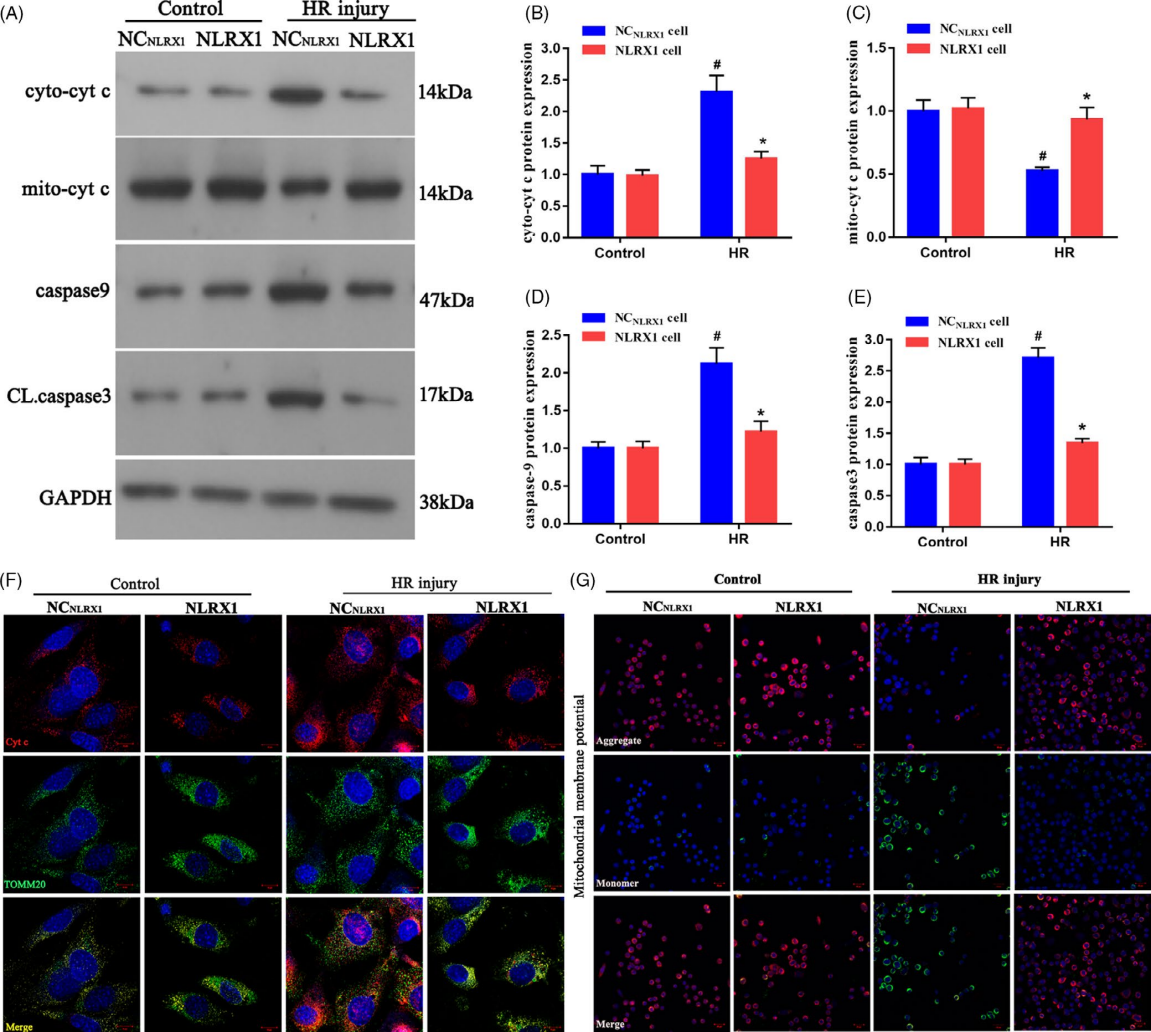
NLRX1 alleviates the damage to mitochondria under intestinal HR in vitro. A‐E, Western blots were used to analyse the mitochondrial apoptotic proteins with or without NLRX1 overexpression. F, The co‐localization of cyt‐c and mitochondria in IEC‐6 cells. (scale bar = 10 μm; magnification ×600). G, Mitochondrial potential as observed via JC‐1 staining (scale bar = 20 μm; magnification ×200). *^#^P *< .05 vs control group; *P < .05 vs NC_NLRX1_+HR group. Cyto‐cyt, cytoplasmic cytochrome‐*c*; mito‐cyt, mitochondrial cytochrome‐c; CL, caspase 3, cleaved caspase 3

We further were interested to identify the roles of NLRX1 in relation to mitochondrial membrane stability. Using the in vitro IEC‐6 cells, we observed that after HR injury, cytoplasmic cytochrome‐c (cyto‐cyt‐*c*) levels were significantly increased whereas mitochondrial cyt‐*c* (mito‐cyt‐*c*) were decreased (Figure 5A‐C). However, after overexpression of NLRX1, cyto‐cyt‐*c* and mito‐cyt‐*c* significantly recovered similar to that of the control. The increase in the levels of cyto‐cyt‐*c* could indicate a potential increase in mitochondrial membrane leakage and apoptosis under HR injury. Further, we also observed a significant increase in caspase‐9 and cleaved caspase 3 levels post‐HR injury. Interestingly, all of these markers could be significantly decreased after NLRX1 overexpression, which indicated a potential rescue from HR‐associated apoptosis (Figure 5A,D,E). Further, using co‐localization experiments, we observed that in control cells cyt‐c could be observed as clear punctate‐like structure and co‐localized with mitochondrial marker translocase of outer mitochondrial membrane 20 (TOMM20) (Figure 5F). However, HR injury resulted in an increased dispersion of the cyt‐*c* protein to both the cytoplasm and the nucleus, which indicates an increase in permeability of the mitochondrial membrane. Interestingly, we show that NLRX1 overexpression could significantly rescue this defect. Further, we assessed the mitochondrial membrane potential using JC‐1 probe. Our results showed that HR injury had very low JC‐1‐associated red fluorescence but increased green fluorescence thus indicating a collapsed mitochondrial membrane potential and unhealthy apoptotic mitochondria (Figure 5G). Alternatively, NLRX1 overexpression increased the JC‐1 red fluorescent signal, thus indicating a rescue of the mitochondria from HR‐associated deleterious effect. These results indicate that NLRX1 inhibits the negative effects of HR injury by decreasing mitochondrial‐associated apoptosis.

### 3.6 NLRX1 inhibits apoptosis by inducing mitophagy in HR injured cells

**FIGURE 6:**
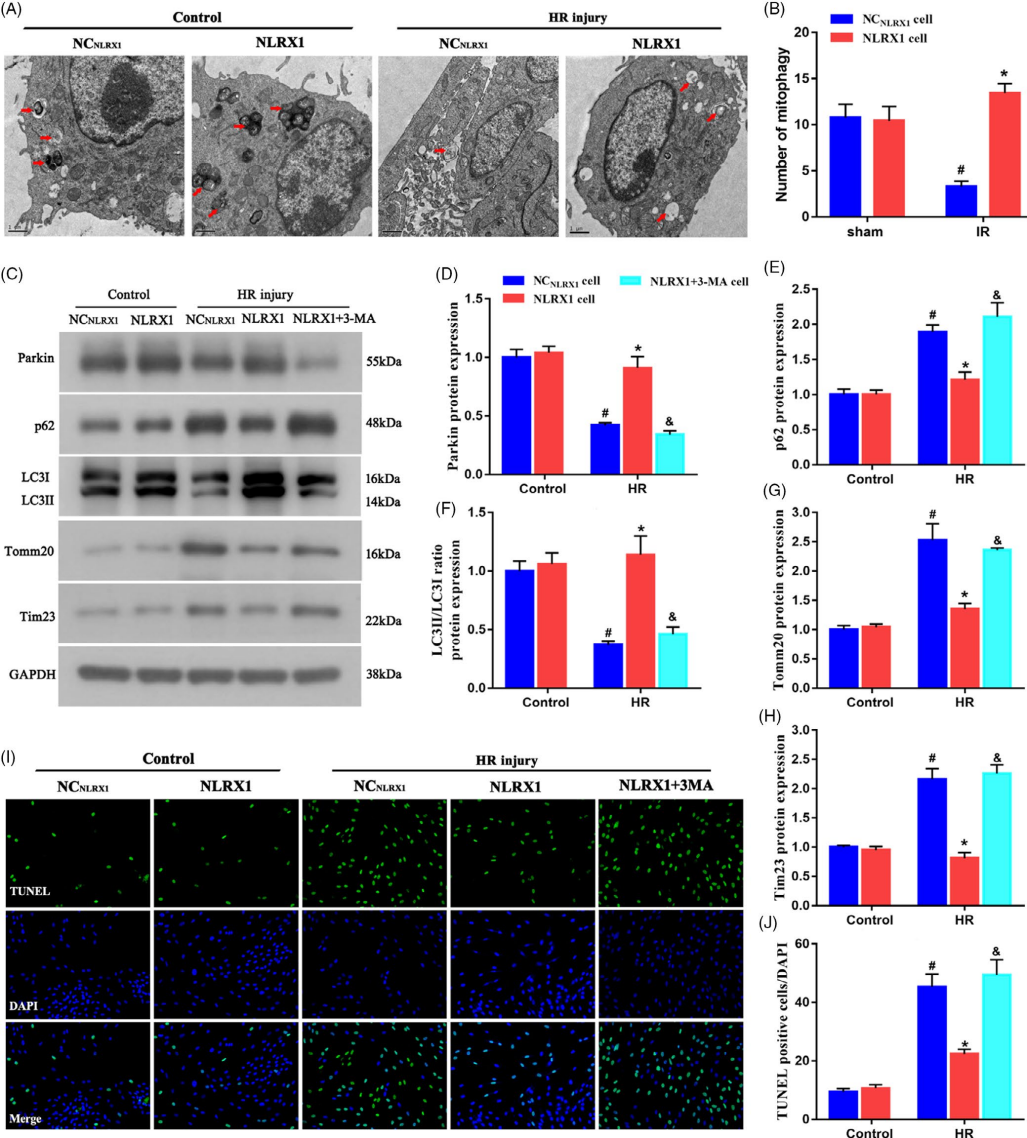
NLRX1 inhibits apoptosis by inducing mitophagy in HR injured cells. A,B Representative transmission electron microscopy images and quantitative analysis of autophagosomes following HR in IEC‐6 cells. Arrows indicating autophagosomes (scale bar = 1 μm; magnification ×60 000). C‐H, Western blots were used to analyse the mitophagy parameters. I,J, TUNEL assay was used to label the apoptotic cells. *^#^P* < .05 vs control group; *P < .05 vs NC_NLRX1_‐cell+HR group; ^&^ *P* < .05 vs NLRX1‐cell+HR group

Next, using transmission electron microscopy (TEM), we observed that the HR injury decreased mitophagy levels as indicated by the decreased number of autophagosomes in IEC‐6 cells (Figure 6A,B). However, NLRX1 overexpression seems to significantly increase autophagosome levels. Additionally, when we performed Western blotting, it was evident that HR injury decreased Parkin levels, similar to our in vivo observations. In addition, overexpression of NLRX1 increased Parkin levels, indicating that indeed NLRX1 overexpression increases mitophagy in IEC cells. Additionally, we used a mitophagy inhibitor (3‐MA) on cells overexpressing NLRX1 and observed that Parkin levels were indeed decreased after the use of 3‐MA even in the presence of NLRX1. Further, we observed that HR injury increased p62 and decreased LC3‐II, and however, overexpression with NLRX1 decreased p62 and increased LC3‐II. Alternatively, use of 3‐MA in NLRX1 overexpressed cell reversed this effect. Further, we also observed mitochondrial markers (Tomm20 and Tim23) to be highly expressed under HR injury, which could be reversed with overexpression of NLRX1. However, 3‐MA treatment again reversed the effect of NLRX1 in the cell model (Figure 6C‐H). These results further strengthened our hypothesis that NLRX1 induced mitophagy in the HR‐induced cell model. Next, we performed TUNEL staining and observed that HR injury did increase apoptosis, and NLRX1 overexpression decreased apoptosis levels. In addition, use of 3‐MA increased the apoptosis levels, indicating that indeed NLRX1 decreased apoptosis levels by regulating the mitophagy levels (Figure 6I,J). These results thus confirmed that NLXR1 inhibits apoptosis by inducing mitophagy in HR‐induced cells.

### 3.7 Fundc1 deletion represses NLRX1‐mediated mitophagy

**FIGURE 7:**
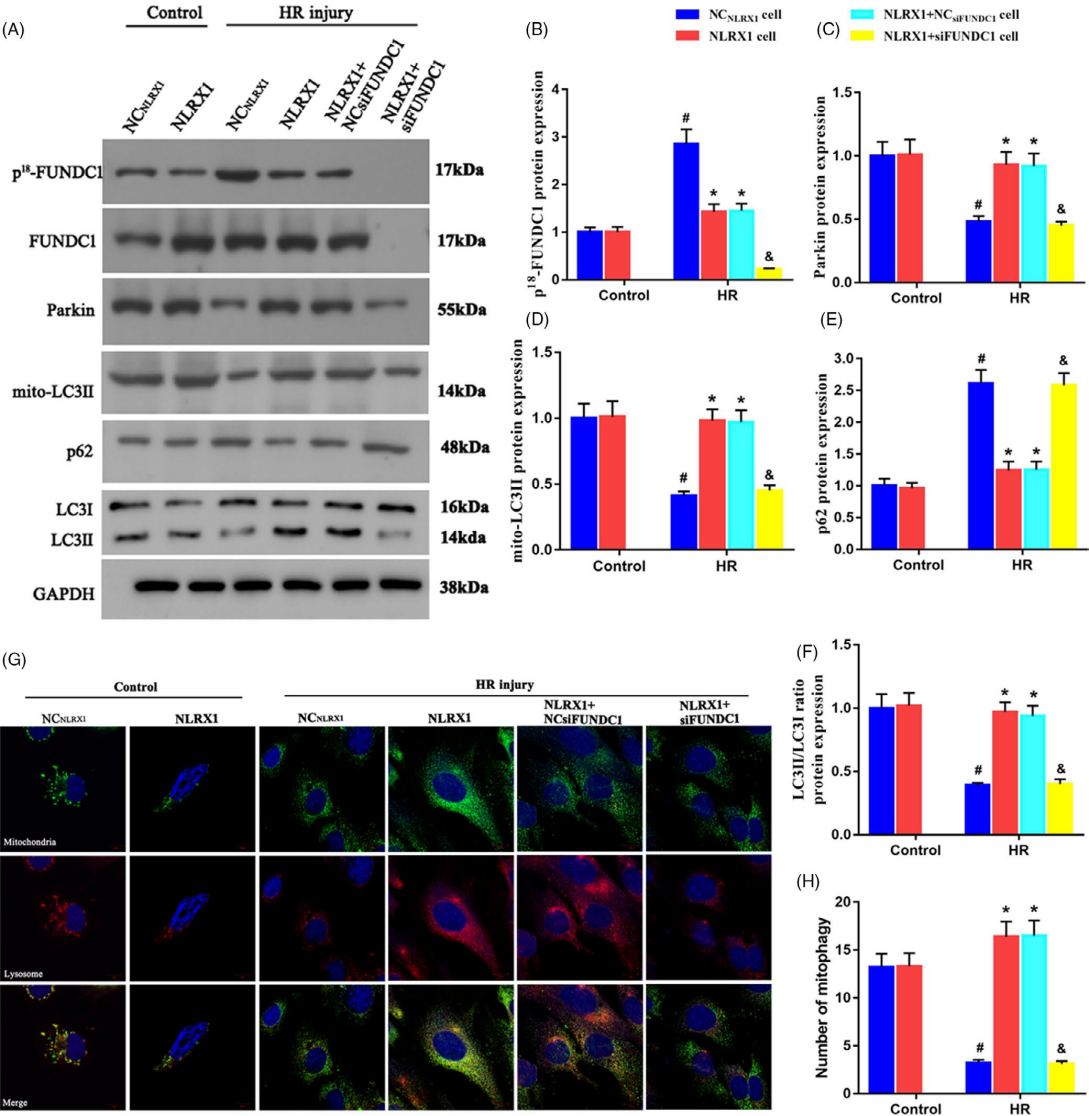
Fundc1 deletion represses NLRX1‐mediated mitophagy in HR injury. A‐F, Western blotting experiments to assess the levels the mitophagy markers. FUNDC1 siRNA was used to silence the FUNDC1 expression. G‐H, Mitophagy activity was observed via co‐staining mitochondria and lysosome at the same time (scale bar = 10 μm; magnification ×600). *^#^P* < .05 vs control group; *P < .05 vs NC_NLRX1_‐cell+HR group; ^&^ *P* < .05 vs NLRX1‐cell+HR group

As we wanted to further elucidate the association between FUNDC1 and HR injury, we developed a FUNDC1 silenced IEC‐6 cells line. Initially, we confirmed the lack of FUNDC1 expression using Western blotting (Figure 7A,B). Further, we assessed the levels of parkin protein, which is a key marker associated with induction of autophagy in mitochondria. Initially, in HR injury model we observed a very less expression level of parkin when compared to the control groups (Figure 7A,C). However, this decrease in parkin levels could be recovered with overexpression of NLRX1. These results further confirmed the role of NLRX1 in mitophagy. Interestingly, silencing of FUNDC1 significantly decreased parkin levels even with the overexpression of NLRX1. We further checked other key autophagy proteins such as mito‐LC3II, p62, LC3I and LC3II levels in FUNDC1‐silenced cells. Similar to parkin levels, mito‐LC3II and LC3II levels were significantly decreased in the presence of HR injury (Figure 7A,D). Evidentially, p62 levels were increased in the presence of HR injury (Figure 7A,E). These results clearly indicated an inhibition in the upstream autophagy process or a potential degradation of auto‐phagolysosome processes. However, overexpression of NLRX1 could significantly rescue this defect and thus could increase the LC3II levels, which confirmed our previous results. Alternatively, it is important to note that silencing of FUNDC1 significantly decreased LC3II levels even in the presence of NLRX1 (Figure 7A,D,F). These results thus confirmed that FUNDC1 is required for NLRX1’s rescue of HR reduced mitophagy. Further, we performed co‐localization experiments where we observed a decrease in lysosomal levels and a further decrease in co‐localization with mitochondrial structures under HR injury (Figure 7G,H). However, NLRX1 overexpression rescued this defect and displayed an increased mitochondrial lysosomal fluorescence signal. Similar to the previous results, however, lack of FUNDC1 made the NLRX1’s rescue of mitophagy ineffective, thus indicating the importance of FUNDC1 in HR injury. Hence, these results clearly indicate that FUNDC1 is essential for NLRX1 mediated rescue of HR injury.

### 3.8 FUNDC1‐required mitophagy sustained mitochondrial homeostasis and favoured cell survival under HR injury

**FIGURE 8:**
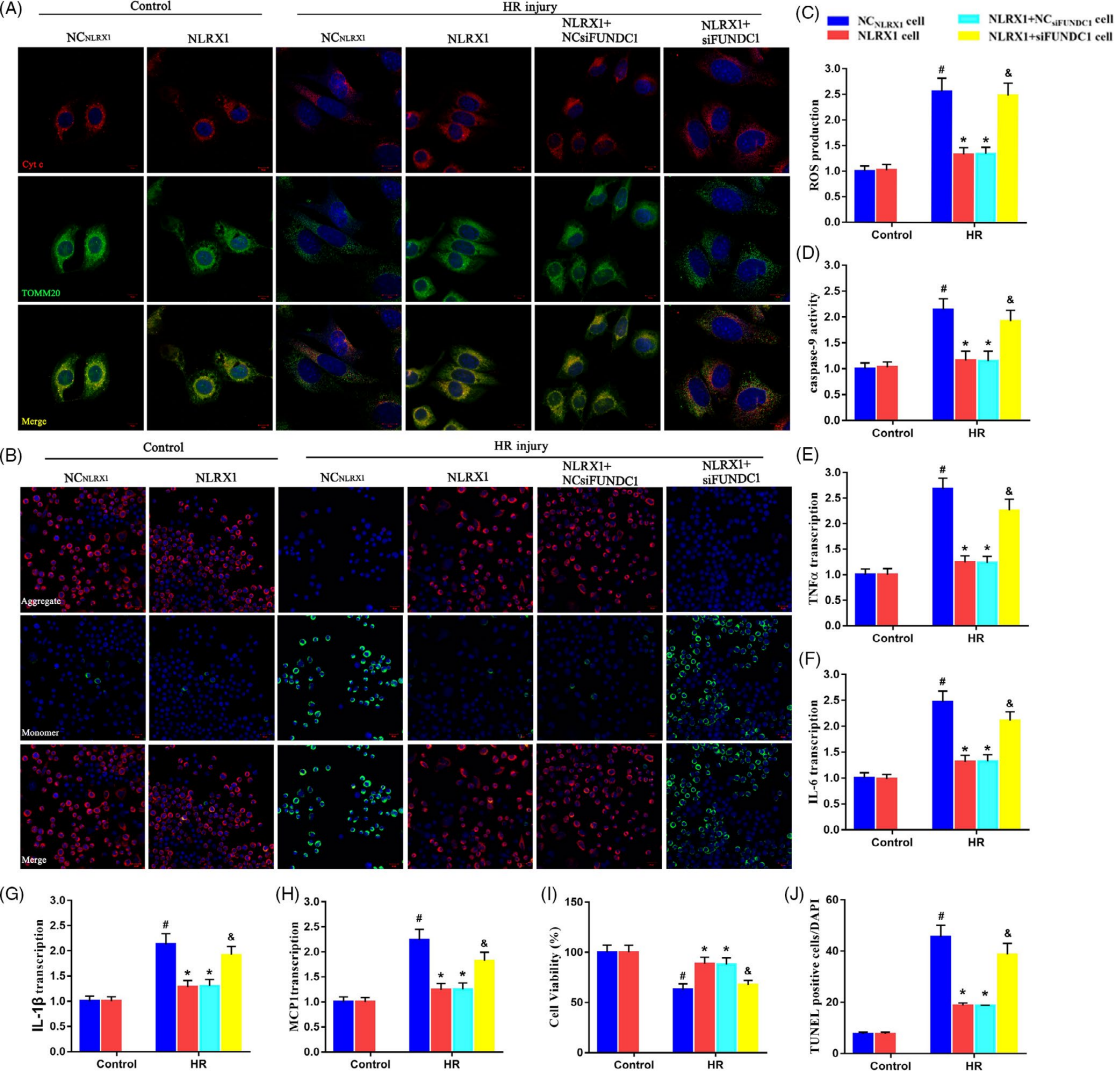
FUNDC1‐required mitophagy sustained mitochondrial homeostasis and favoured cell survival under HR injury. A, The cyt‐c leakage from mitochondria into the cytoplasm/nuclear area was observed via immunofluorescence assay. Increased mitophagy was associated with less cyt‐*c* diffusion into the cytoplasm in IEC‐6 cells (scale bar = 10 μm; magnification ×600). B, JC‐1 staining was used to observe the mitochondrial membrane potential (scale bar = 20 μm; magnification ×200). C, The cellular ROS was measured using DCFDA assay. D, The transcriptional alteration in apoptotic factor caspase‐9. E‐H, The transcriptional alteration in inflammatory factors TNFα, IL‐6, IL‐1β and MCP1. I, Cell viability was examined using CCK‐8 assay. J, Representative TUNEL quantitative analysis after HR injury. Relative apoptotic rates are presented as TUNEL‐positive cells/DAPI. *^#^P* < .05 vs control group; *P < .05 vs NC_NLRX1_‐cell+HR group; ^&^ *P* < .05 vs NLRX1‐cell+HR group

We further wanted to understand the role of FUNDC1 on the maintenance of mitochondrial homeostasis and membrane potential. Hence, we assessed the leakage of cyto‐*c* from the mitochondria in the presence and absence of FUNDC1. As mentioned previously, HR injury increased the leakage of cyt‐*c* from the mitochondria into the cytoplasm, which could be recovered using NLRX1 overexpression (Figure 8A). However, silencing of FUNDC1 even in the presence of NLRX1 overexpression seems to increase the leakage of cyt‐*c* from mitochondria. These results indicate that FUNDC1 and NLRX1 are required for maintaining the cyt‐*c* levels in the mitochondria. In addition, we assessed the defects in membrane potential and observed that HR injury decreases the membrane potential as indicated by the increase in green fluorescence (Figure 8B). However, NLRX1 overexpression rescued this defect as shown by the JC‐1 staining. Further, lack of FUNDC1 completely negates this effect of NLRX1 overexpression, thus indicating that FUNDC1 is necessary to maintain the mitochondrial membrane potential. Next, we checked the ROS levels and it was evident that HR injury increased ROS levels, which could be decreased again using NLRX1 overexpression (Figure 8C). Alternatively, lack of FUNDC1 seems to reverse this effect and increase the ROS levels again. This was further evident with other experiments where we assessed the transcription activity of inflammatory factors such as TNFα, IL‐6, IL‐1 β and MCP1 (Figure 8E‐H). From these assays, it was evident that increase in inflammatory factors post‐HR injury could be rescued by NLRX1 overexpression, however only in the presence of FUNDC1. Additionally, from the cell viability CCK‐8 assays, we observed that HR injury decreased cell viability (Figure 8I). Again, NLRX1 overexpression could increase and recover the cell viability rates, but in the absence of FUNDC1, NLRX1 could not efficiently function in recovering the cell viability. Apoptotic rates using TUNEL and caspase‐9 also indicated that NLRX1 could rescue and decrease the HR injury‐induced apoptosis, in a FUNDC1‐dependent manner (Figure 8D,J).

### 3.9 Overexpressed NLRX1 activates the NIPSNAP1‐2 ‘eat me’ signalling for mitophagy by inhibiting phosphorylation of FUNDC1

**FIGURE 9:**
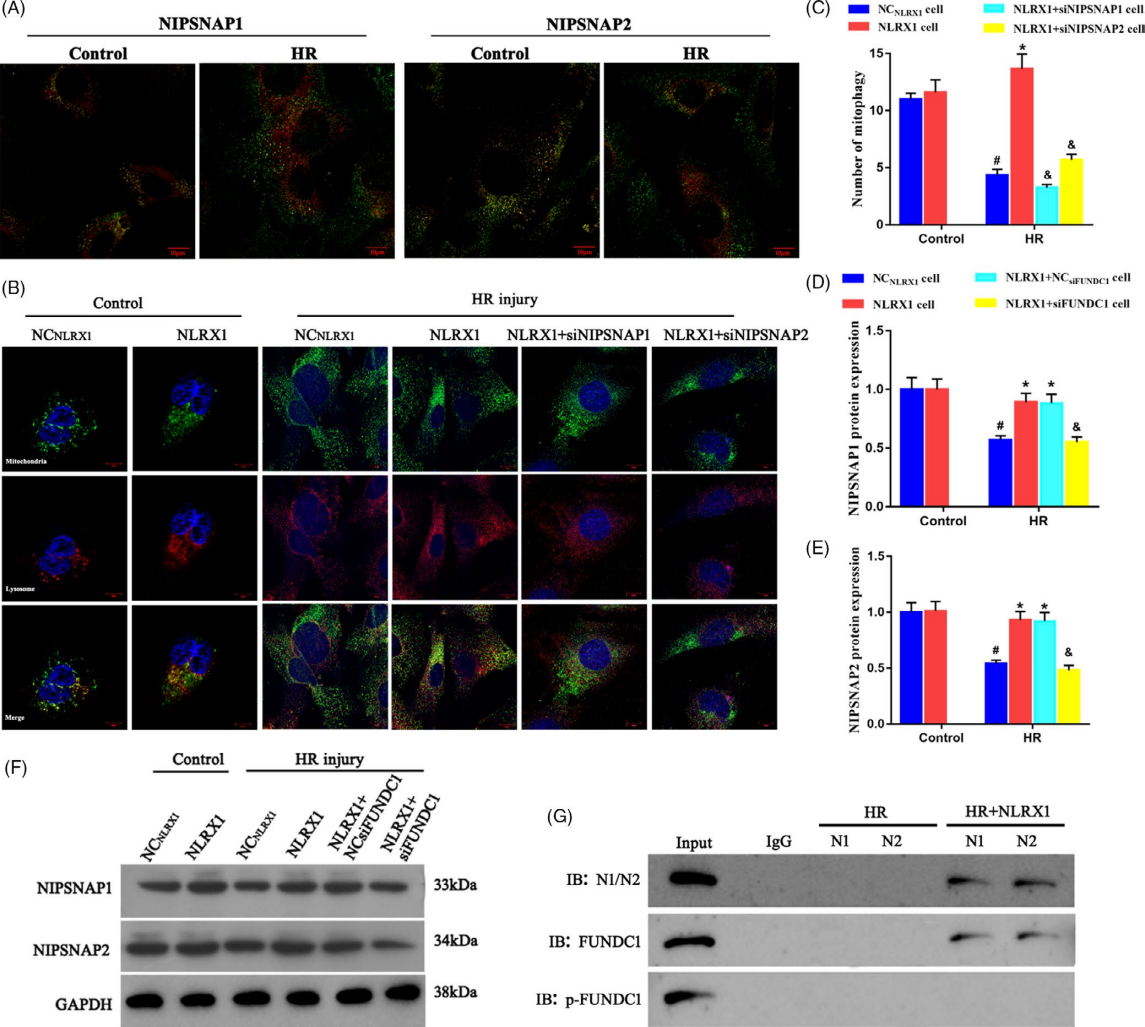
Overexpressed NLRX1 activates the NIPSNAP1‐2 signalling by inhibiting phosphorylation of FUNDC1. A, NIPSNAP1 and NIPSNAP2 accumulate on the mitochondrial outer membrane following HR. Red: NIPSNAP1 or NIPSNAP2; Green: mitochondria. B‐C, The co‐immunofluorescence of lysosome and mitochondria in IEC‐6 cells (scale bar = 10 μm; magnification ×600). D‐F, Western blots were used to analyse NIPSNAP1 and NIPSNAP2. G, NLRX1 and FUNDC1 interaction was assessed by immunoprecipitation (IP) experiments. N1: NIPSNAP1; N2: NIPSNAP2. *^#^P* < .05 vs control group; *P < .05 vs NC_NLRX1_‐cell+HR group; ^&^ *P* < .05 vs NLRX1‐cell+HR group

Finally, to identify the correlations between NLRX1, FUNDC1 and mitophagy, we assessed the levels of NIPSNAP1‐2 under HR injury. Initially, using immunofluorescence staining, we observed that NIPSNAP1‐2 accumulated in the mitochondrial outer membrane under HR injury (Figure 9A). Additionally, we performed co‐immunofluorescence experiments with lysosome and mitochondrial antibody and identified that HR injury in these cells decreased the co‐localization and hence decreased mitophagy. However, overexpression with NLRX1 significantly seems to increase the mitophagy levels. Further, when we overexpressed NLRX1 and silenced NIPSNAP1/2, we observed an opposite effect. That is, we observed that absence of NIPSNAP1/2 reversed the effect of NLRX1 on HR injury (Figure 9B,C). Further, using Western blotting, we observed that there was actually a decrease in NIPSNAP1‐2 levels under HR injury (Figure 9F). As indicated before, NLRX1 overexpression increased the NIPSNAP1‐2 levels, but only in the presence of FUNDC1 (Figure 9D‐F). Further, using immunoprecipitation experiments, we observed that there was no FUNDC1 bound to NIPSNAP1‐2 after HR injury. However, NLRX1 overexpression after HR injury could increase the levels of FUNDC1 bound to NIPSNAP1‐2 (Figure 9G). Interestingly, there was no p‐FUNDC1 bound to NIPSNAP1‐2 after HR or after NLRX1 overexpression. These results indicated that FUNDC1 in its unphosphorylated form binds and activates NIPSNAP1‐2 to allow activation of mitophagy. Additionally, NLRX1 inhibits the phosphorylation of FUNDC1 that thus increases mitophagy and decreases apoptosis of cells affected by HR injury.

## 4 DISCUSSION

**FIGURE 10:**
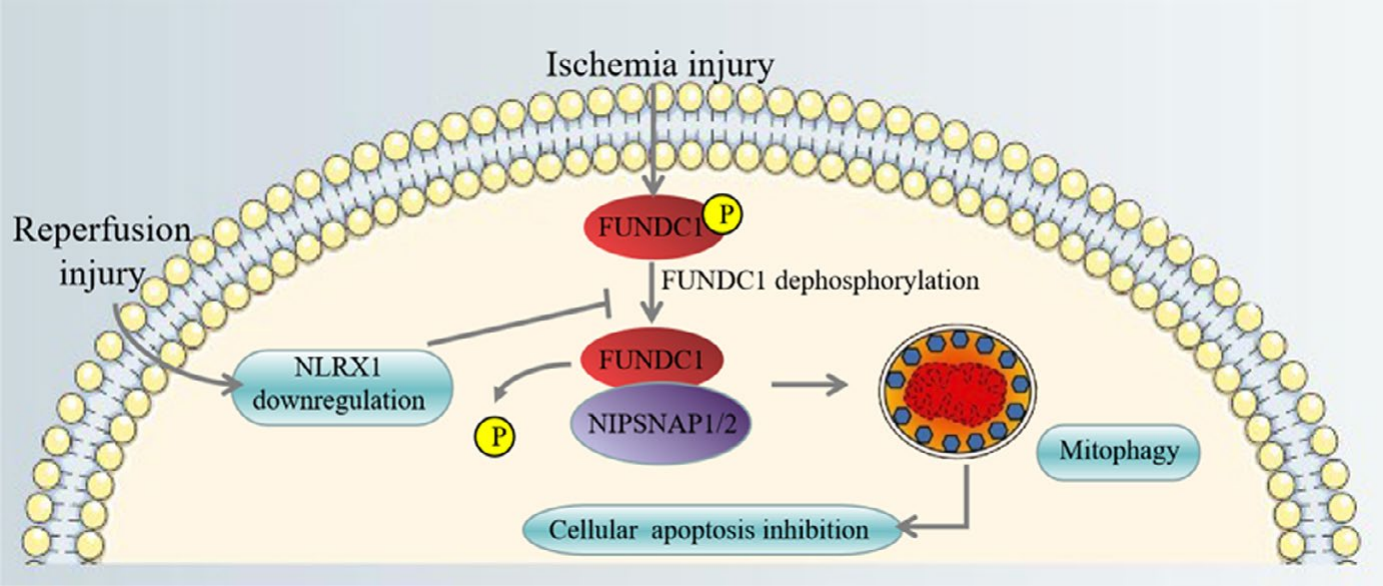
Under ischaemia, FUNDC1 was activated and interacted with NIPSNAP1 and NIPSNAP2, facilitated mitophagy to selectively degrade the doomed mitochondria and reduce pro‐apoptotic factor cyt‐*c* leakage into the cytoplasm, eventually inhibiting caspase‐9‐involved apoptosis. However, after reperfusion, NLRX1 was downregulated, contributing to FUNDC1 deactivation via the post‐transcriptional modification of the FUNDC1 phosphorylation site. Phosphorylated FUNDC1 cannot be interacted with NIPSNAP1 and NIPSNAP2, which failed to launch the protective mitophagy process, resulting in the accumulation of damaged mitochondria and epithelial apoptosis

IR injury has been strikingly associated with high mortality and morbidity.^29^, ^30^ Further, IR injury of the intestine could lead to an impairment of the intestinal barrier, thus causing higher permeability and change in the intestinal microbial environment. This further could lead to severe inflammatory response and end in organ failure.^6^, ^7^ Due to the above‐mentioned reasons, there is an increasing need to develop diagnostic and therapeutic strategies to treat intestinal IR injury. In this study, we initially identified NLRX1 is significantly downregulated after intestinal IR injury (Figure 1A). Interestingly, overexpression of NLRX1 mitigated the effects of IR injury through the increase of mitophagy and decrease in apoptosis (Figure 3C,E). We further identified that NLRX1 alleviates intestinal IR injury through the regulation of FUNDC1‐NIPSNAP1/NIPSNAP2 axis in intestine (Figure 10).

Previously, studies have identified that the deleterious effects of myocardial, hepatic and intestinal IR injury could be associated with mitochondrial dysfunction.^9^, ^10^, ^31^ Classically, increased free radical production by the mitochondria has been assumed as the major cause for the damage caused to the organs.^32^ However, recently, studies have confirmed that there are other more complex players such as mitochondrial dysfunction that originally acts as a cause for this disease.^9^, ^10^, ^31^ As previously mentioned, mitophagy is the cellular process by which damaged mitochondria are recycled thus preventing accumulation of toxic products and damaged mitochondria.^9^, ^10^ And a defect in mitophagy leads to the accumulation of toxic products, collapse in mitochondrial membrane potential and ultimately cell death.^33^ Mitochondrial dysfunction have been associated with neurodegenerative diseases, diabetes and ageing.^34^, ^35^, ^36^ Interestingly, this mitochondrial dysfunction is caused by vicious cycle of multiple incidents. First, lack of blood supply to organs due to ischaemia decreases the availability of ATP and nutrient depletion.^37^ Further, in IR hepatic ischaemia, this lack of ATP contributes to a shutdown of ATP‐driven calcium channels which leading to an increase in accumulation of intercellular calcium and ultimately causes mitochondrial permeability transition (MPT). Further, MPT induces depolarization of the mitochondrial membrane thus contributing to apoptotic cell death.^37^ Crucially, both autophagy and apoptosis play a balancing role during IR injury. Previously, IR studies have indicated that autophagy induced under stressful conditions allow protection by inhibiting apoptosis.^38^, ^39^ This activation of autophagy in IR seems to inhibit apoptosis by clearing up misfolded protein or damaged mitochondria.^40^ Damaged mitochondria in turn could release pro‐apoptotic factor cyt‐*c* into the cytoplasm, trigger apoptosome formation (a complex comprising Apaf‐1 cyt‐*c*, dATP and pro‐caspase‐9) and caspase‐9‐related mitochondrial apoptosis activation that in turn activates pro‐caspase‐3 to form the cell death effector cleaved caspase‐3.^41^, ^42^ In our study, we observed that intestinal IR injury causes a collapse in membrane potential and increase in apoptosis, and however, overexpression of NLRX1 increased membrane potential and decreased apoptosis (Figure 5G). Secondly, rupture of mitochondrial membrane due to IR injury leads to an increase in leakage of cytochrome‐c from mitochondria to the cytosol, which triggers caspase‐ or ATP‐dependent apoptosis.^43^ In our study, we also observed an increase in leakage of cytochrome‐c from the mitochondria to the cytosol; however, overexpression of NLRX1 decreased the leakage of cytochrome‐*c* (Figure 5F). Thirdly, lack of ATP in ischaemic liver has shown to increase calcium‐dependent caspase activity along with hydrolyzation of important autophagy proteins such as ATG7 and BECN1. In our study, we observed decreased caspase‐9 and cleaved caspase 3 after overexpression with NLRX1 (Figure 5A). Hence, in our study, we observed that NLRX1 overexpression decreased ROS and inflammatory marker production, decreased apoptosis, mitigated the leakage of cytochrome‐*c* from mitochondria to cytosol and increased mitophagy. These results indicate that NLRX1 is a key candidate for the treatment of IR injury. However, there are very few studies that have assessed the role of NLRX1 in IR injury. A study by Stokman et al. identified that NLRX1 decreases apoptosis and ROS production in renal‐IR injury, but did not further explore on the mechanisms behind NLRX1’s role in IR injury.^44^ Another study identified that post‐mitochondrial injury, NLRX1 interacts with inflammatory mediators such as IKKβ and NF‐κB and reduced innate immune response and apoptosis.^45^ In our study, we further extended the understanding of the mechanism behind NLRX1’s role on intestinal IR injury.

Initially, we identified that decrease in NLRX1 in HR injury in vitro models increased the levels of phosphorylated Tyr 18 FUNDC1 levels (Figure 4E). Previously, mammalian STE20‐like kinase 1, an enzyme identified to be highly expressed after cardiac IR injury have been found to downregulate the expression of FUNDC1 and thus inhibit mitophagy and increase apoptosis.^31^ In our study as well, decreased FUNDC1 levels, that is, increased phosphorylated Tyr 18 FUNDC1 levels, also seem to increase apoptosis through decreased mitochondrial membrane potential and increased leakage of cytochrome‐*c* (Figure 8A,B). Additionally, siFUNDC1 in our HR injury cell model decreased the levels of autophagic markers such as Parkin (Figure 7A). NLRX1 overexpression seems to decrease the phosphorylation of FUNDC1, which rescues the mitochondria through increase in mitophagy and decrease of apoptosis. Interestingly, previous studies have also indicated that an NLRX1 overexpression can decrease phosphorylation of many key immune regulators such as IKKβ and NF‐κB.^46^ However, the exact mechanism by which NLRX1 modifies FUNDC1 phosphorylation remains unclear, and more research is required to further understand the mechanism behind it. NIPSNAP1/NIPSNAP2 have been previously identified to interact with key autophagic proteins such as p62 and ATG and thereby regulating the autophagy of damaged mitochondria. Further, studies indicate that a collapse of the membrane potential seems to increase the proportion of NIPSNAP1 in the mitochondrial surface. This accumulation contributes to an increased recruitment of autophagic proteins.^47^ However, in our study, we observed IR injury led to a decreased NIPSNAP1/2 levels, and this could be correlated with increased phosphorylated FUNDC1 levels (Figure 9F). This connection between FUNDC1 and NIPSNAP1/2 is further evident through previous literature where NIPSNAP1 and NIPSNAP2 participate in the regulation of mitochondrial autophagy by recruiting mitochondrial autophagy receptors, and FUNDC1 as a mitochondrial autophagy receptor can also regulate mitochondrial autophagy.^27^, ^47^ Using co‐immunoprecipitation studies, we found that NLRX1 overexpression after HR injury could increase the levels of FUNDC1 bound to NIPSNAP1‐2, which could explain the increase in autophagy levels in the mitochondria (Figure 9g).

In this study, we have identified the role of NLRX1‐FUNDC1‐NIPSNAP1/NIPSNAP2 axis in activating mitochondrial autophagy as a rescue mechanism against IR injury. Thus, NLRX1 could be a key target for the development of potential treatment strategies against intestinal IR injury.

## CONFLICT OF INTEREST

The authors declared no conflict of interest in this manuscript.

## AUTHOR CONTRIBUTIONS

SL, YZ and XG conceived the idea and designed the experiments; SL, YZ and XG led the experiments (with assistance from XZ and ZJ); SL, YZ, XZ and ZJ contributed to data analysis and interpretation; YZ and XG wrote the paper; XZ and ZJ supervised the entire project. All authors read and approved the final manuscript.

## Data Availability

The data that support the findings of this study are available from the corresponding author upon reasonable request.
